# Unplanned 3-day re-attendance rate at Emergency Department (ED) and hospital’s bed occupancy rate (BOR)

**DOI:** 10.1186/s12245-015-0082-3

**Published:** 2015-08-25

**Authors:** Yan Sun, Bee Hoon Heng, Seow Yian Tay, Kelvin Brian Tan

**Affiliations:** Department of Health Services & Outcomes Research, National Healthcare Group, 3 Fusionopolis Link, #03-08 Nexus@one-north, 138543 Singapore; Department of Emergency Medicine, Tan Tock Seng Hospital, 11 Jalan Tan Tock Seng, 308433 Singapore; Policy Research & Economics Office, Ministry of Health, 16 College Road, 169854 Singapore

## Abstract

**Background:**

Unplanned re-attendance at the Emergency Department (ED) is often monitored as a quality indicator of the care accorded to patients during their index ED visit. High bed occupancy rate (BOR) has been considered as a matter of reduced patient comfort and privacy. Most hospitals in Singapore operate under BORs above 85 %. This study aims to explore factors associated with the unplanned 3-day ED re-attendance rate and, in particular, if higher BOR is associated with higher 3-day unplanned ED re-attendance rate.

**Methods:**

This was a multicenter retrospective study using time series data. Three acute tertiary hospitals were selected from all six adult public hospitals in Singapore based on data availability. Daily data from year 2008 to 2013 were collected from the study hospitals’ information systems. These included: ED visit date, day of week, month, year, public holiday, daily hospital BOR, daily bed waiting time (BWT) at ED (both median and 95th percentile), daily ED admission rate, and 3-day ED re-attendance rate. The primary outcome of the study was unplanned 3-day ED re-attendance rate from all reasons. Both univariate analysis and generalized linear regression were respectively applied to study the crude and adjusted association between the unplanned 3-day ED re-attendance rate and its potential associated factors. All analyses were conducted using SPSS 18 (PASW 18, IBM).

**Results:**

The average age of patients who visited ED was 35 years old (SD = 2), 37 years old (SD = 2), and 40 years old (SD = 2) in hospitals A, B, and C respectively. The average 3-day unplanned ED re-attendance rate was 4.9 % (SE = 0.47 %) in hospital A, 3.9 % (SE = 0.35 %) in hospital B, and 4.4 % (SE = 0.30 %) in hospital C. After controlling for other covariates, the unplanned 3-day ED re-attendance rates were significantly associated with hospital, time trend, day of week, daily average BOR, and ED admission rate. Strong day-of-week effect on early ED re-attendance rate was first explored in this study. Thursday had the lowest re-attendance rate, while Sunday has the highest re-attendance rate. The patients who visited at ED on the dates with higher BOR were more likely to re-attend the ED within 3 days for hospitals A and B. There was no significant association between BOR and ED re-attendance rate in hospital C.

**Conclusions:**

A study using time series data has been conducted to explore the factors associated with the unplanned 3-day ED re-attendance rate. Strong day-of-week effect was first reported. The association between BOR and the ED re-attendance rate varied with hospital.

## Background

Re-attendance at the Emergency Department (ED) is a measure of the level of appropriate care given to patients during their preceding ED visit [1, 2], with unscheduled return to the ED within 72 h as one of the common indicators. This may represent premature discharges from the first ED visit, missed diagnosis, or a failure in the treatment or discharge plan [3, 4]. Rates above 5 % may suggest poor quality, while rates below 1 % may reflect excessive risk aversion [5, 6].

Patients who return to ED fall into patient-related factors (gender, age, socioeconomic status, insurance status, inability to understand or comply with discharge planning, and misuse of emergency services), illness-related factors (worsening of an existing condition, acute exacerbation of a chronic condition, complications arising from disease, and new health problems), health-care staff or hospital-related factors (misdiagnosis, malpractice, inadequate communication between health-care providers and patients, and lack of subsequent referral services or continuity of care were identified as major issues), and many other reasons [7–10]. It is also of great interest for hospital administrators to know how daily operational factors (like bed occupancy rate (BOR), patients’ BWT, admission rate, etc.) affect the unplanned 3-day ED re-attendance rate to help them monitor and improve hospital operations [11, 12].

High BOR is a problem for hospitals in many countries worldwide [13–18]. Hospitals with BORs above 85 % are generally considered to have bed shortages associated with reduced patient comfort, privacy, and safety [14–16, 18–20]. Most hospitals in Singapore operate under BORs above 85 % according to statistics provided by Ministry, Singapore [21]. High BOR may also cause delayed patient flow from ED to inpatient wards, resulting in overcrowding and resource constraints in ED [22, 23] as more ED resources would be allocated to take care of those patients waiting at ED.

Little attention has been paid to the impact of the “crowdedness” or resource constraints caused by high BOR on ED service performance or ED patients’ outcomes. As far as we know, there is only one recently published study testing the hypothesis if higher BOR causes higher 3-day unplanned ED re-attendance rate. It is still unclear what factors might affect the ED re-attendance rate on a daily operational basis [24]. To address this gap, this study aims to determine (1) the daily factors (like BOR, BWT, admission rate, day of week or public holiday, etc.) associated with the 3-day ED re-attendance rate and especially (2) if higher BOR is associated with higher 3-day unplanned ED re-attendance rate.

## Methods

### Design

This was a multi-center retrospective study using time series data. Ethics review was approved by the Institutional Review Board (IRB) of the National Healthcare Group. There were no personal patient data collected in this study.

### Setting

Three acute tertiary hospitals were selected from all six public hospitals in Singapore based on data availability. Hospital A has about 360 beds, hospital B has about 900 beds, and hospital C has about 1300 beds. Hospital C serves the highest number of ED attendances among all public general hospitals in Singapore.

### Data

Data from 2008 to 2013 were extracted from information systems of the three selected hospitals. The information included the following: ED visit date, day of week, month, year, public holiday, daily hospital BOR, average daily BWT at ED (both median and 95^th^ percentile), daily ED admission rate, and 3-day ED re-attendance rate. A variable of time trend was generated from the ED visit date to indicate the collected time points, with the first study day (January 1, 2008) as 1, and the last study day (December 31, 2013) as 2192. The primary outcome of the study was the unplanned 3-day ED re-attendance rate from all causes. All data were measured at daily level. The definitions of these measurements are listed as follows:

**Hospital BOR**: percent of beds occupied among all beds based on the midnight bed census at each hospital (for example, the BOR for Monday is based on the bed census taken at 0000 h Tuesday)

**ED admission rate**: percent of patients admitted to inpatient wards among those who visited ED

**Unplanned 3-day ED re-attendance rate**: percent of patients with an unplanned re-attendance to any ED within 3 days among all those who visited and were discharged from the ED

**Bed waiting time**: time from requesting a bed at ED to admission to inpatient ward

### Statistical analysis

ANOVA test was applied to study the association between the unplanned 3-day ED re-attendance rate and its associated factors for categorical variables; while bivariate correlation analysis was applied for scale variables. Generalized linear models (GLM) were applied to study the adjusted association between the outcome and the potential association factors. Maximum-likelihood estimation method was used in GLM for estimating the risk ratios and their confidence intervals. The outcome was nearly normally distributed. A model with gamma distribution and log transform was compared with a model with normal distribution and no transform for fitting the observed outcome. Best fit model was selected using Bayesian information criteria (BIC) and Pearson chi-square statistics. All analyses were conducted using SPSS 18 (PASW 18, IBM).

## Results

The average age of the patients who visited ED was 35 years old (SD = 2), 37 years old (SD = 2), and 40 years old (SD = 2) in hospitals A, B, and C, respectively. The median age for the three hospitals was 36, 37, and 40 years old, respectively.

**Fig. 1:**
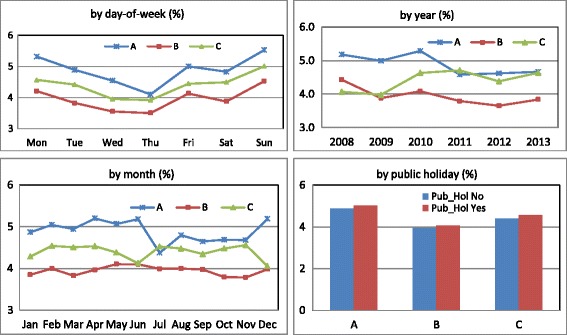
Plot of unplanned 3-day ED re-attendance rates in three hospitals by index ED attendances (*day of week, year, month,* and *public holiday*)

The average unplanned 3-day ED re-attendance rate was 4.9 % (SE = 0.47 %) in hospital A, 3.9 % (SE = 0.35 %) in hospital B, and 4.4 % (SE = 0.30 %) in hospital C. The rates in the three hospitals were significantly associated with day of week, year, month, and public holiday. The highest re-attendance rate was observed for the patients who visited on Sunday, while lowest re-attendance rate was for the patients who visited on Thursday (*p* < 0.001). The rate on public holiday was slightly higher than non-public holidays in all the three hospitals (*p* < 0.001). There was monthly fluctuation in all the three hospitals with no discernible trend observed. There was a slightly decreasing trend for hospital A (Spearman’s rho = −0.11, *p* < 0.001) and hospital B (Spearman’s rho = −0.10, *p* < 0.001), while a slightly increasing trend for hospital C (Spearman’s rho = 0.16, *p* < 0.001) (Fig. 1).

The average patients’ age, daily BOR, ED admission rate, and bed waiting time were significantly associated with the 3-day ED re-attendance rate. However, the associations varied greatly among hospitals, even with different signs of positive or negative correlation (Table 1). The 95^th^ percentile waiting time was preferred to the median percentile waiting time on predicting the 3-day ED re-attendance rate since it was more significant.

**Table 1:** Associations between 3-day ED re-attendance rate and average patients’ age, daily BOR, ED admission rate, and bed waiting time (BWT)

	Hospital A		Hospital B		Hospital C	
	Corr coeff	*p* value	Corr coeff	*p* value	Corr coeff	*p* value
Average age	−0.02	0.369	−0.01	0.690	0.10	<0.001
BOR	0.04	0.043	−0.03	0.176	−0.02	0.340
Admission rate	−0.09	<0.001	−0.05	0.018	−0.12	<0.001
BWT50	−0.08	<0.001	−0.03	0.171	−0.02	0.398
BWT95	−0.03	0.137	−0.02	0.269	−0.06	0.003

Before running multiple regression, multi-collinearity diagnosis was carried out. As age was highly correlated with time trend, admission rate, and BOR, it was removed from the multiple regression. The multi-collinearity diagnosis between BOR and other covariates showed that the possible collinearity between BOR and bed waiting time or ED admission rate could be neglected since all condition index values were less than 30.

The BIC score and Pearson chi-square statistics (value/*df*) for the model with gamma distribution and log transform were 25,477.6 and 0.149, while those values for the model with normal distribution and no transform were 26,085.3 and 3.009, respectively. Therefore, the model with gamma distribution and log transform was selected to fit the observed data. After controlling for other covariates, the significant factors associated with the 3-day ED re-attendance rate were hospital, day of week, month, BOR, admission rate, and time trend. Hospital A had the highest re-attendance rate, followed by Hospital C, and then Hospital B. Attending ED on Thursday had the lowest re-attendance rate, while attending ED on Sunday had the highest re-attendance rate. The patients who visited at ED on the day with higher BOR or lower ED admission rate were more likely to re-attend ED within 3 days (Table 2).

**Table 2:** Independent factors associated with the unplanned 3-day ED re-attendance rate

Parameter	Risk ratio	95 % Confidence interval		*p* value
		Lower	Upper	
*Hospital [*C]				
A	*1.63*	*1.36*	*1.96*	*<0.001*
B	*0.64*	*0.58*	*0.71*	*<0.001*
*Day of week* ^a^ [Thu]				
Mon	*2.27*	*1.94*	*2.67*	*<0.001*
Tue	*1.71*	*1.46*	*2.01*	*<0.001*
Wed	*1.19*	*1.01*	*1.39*	*<0.001*
Fri	*2.05*	*1.75*	*2.40*	*<0.001*
Sat	*1.82*	*1.55*	*2.13*	*<0.001*
Sun	*3.09*	*2.61*	*3.65*	*<0.001*
*Month* ^a^ [Jan]				
Feb	1.22	0.99	1.51	0.058
Mar	1.10	0.90	1.35	0.366
April	*1.26*	*1.03*	*1.55*	*0.027*
May	1.19	0.97	1.45	0.101
Jun	1.15	0.94	1.41	0.180
Jul	1.00	0.82	1.23	0.992
Aug	1.14	0.93	1.41	0.196
Sep	1.03	0.84	1.27	0.756
Oct	1.03	0.84	1.26	0.786
Nov	1.04	0.85	1.28	0.687
Dec	1.15	0.94	1.41	0.185
Public holiday^a^	1.07	0.84	1.36	0.581
*BOR* ^a^	*1.01*	*1.00*	*1.02*	*<0.001*
*Admission rate* ^a^	*0.99*	*0.98*	*1.00*	*0.028*
BWT (95th percentile)^a^	0.98	0.97	1.00	0.059
*Time trend*	*1.00*	*1.00*	*1.00*	*<0.001*

Significant factors were italicized

^a^Refers to the index visit to ED

The associations between 3-day ED re-attendance rate and BOR, admission rate, or bed waiting time might be varied with hospitals’ available resources and their operation efficiency in ED and inpatient wards. In order to assess the heterogeneity among hospitals, a subgroup analysis by hospital was carried out. For all three hospitals, the unplanned 3-day ED re-attendance rate varied significantly with day of week. There was a slightly decreasing time trend in hospitals A and B, while a slightly increasing trend in hospital C. There was no discernible monthly trend. In hospitals A and B, higher BOR was associated with the higher ED re-attendance rate, while it was not true for hospital C. In hospitals A and C, higher ED admission rate was associated with lower ED re-attendance rate. Bed waiting time did not affect the ED re-attendance rate. Public holiday had no association with 3-day ED re-attendance rate (Table 3).

**Table 3:** Adjusted associations between 3-day ED re-attendance rate and the factors by hospital

	Hospital A			Hospital B			Hospital C		
	Risk ratio	95 % Confidence interval		Risk ratio	95 % Confidence interval		Risk ratio	95 % Confidence interval	
Parameter		Lower	Upper		Lower	Upper		Lower	Upper
**Day of week** ^a^ [Thu]									
Mon	*3.14*	*2.24*	*4.40*	*1.92*	*1.49*	*2.47*	*1.99*	*1.60*	*2.47*
Tue	*2.11*	*1.51*	*2.96*	*1.34*	*1.05*	*1.71*	*1.68*	*1.36*	*2.07*
Wed	*1.50*	*1.08*	*2.10*	*1.04*	*0.82*	*1.33*	*1.04*	*0.85*	*1.29*
Fri	*2.56*	*1.83*	*3.58*	*2.07*	*1.61*	*2.66*	*1.64*	*1.32*	*2.02*
Sat	*2.23*	*1.59*	*3.13*	*1.78*	*1.36*	*2.32*	*1.71*	*1.38*	*2.11*
Sun	*4.00*	*2.82*	*5.67*	*2.95*	*2.26*	*3.85*	*3.03*	*2.39*	*3.85*
*Month* ^a^ [Jan]									
Feb	1.21	0.77	1.88	1.18	0.85	1.63	1.27	0.96	1.68
Mar	1.14	0.74	1.76	0.96	0.70	1.32	1.25	0.96	1.64
April	1.42	0.92	2.20	1.12	0.81	1.54	1.29	0.98	1.69
May	1.22	0.79	1.88	1.29	0.94	1.78	1.12	0.85	1.47
Jun	1.39	0.90	2.17	1.37	0.99	1.89	0.83	0.63	1.09
Jul	0.64	0.41	1.00	1.24	0.90	1.71	1.19	0.91	1.56
Aug	1.05	0.68	1.63	1.28	0.93	1.76	1.08	0.82	1.42
Sep	0.92	0.59	1.44	1.25	0.90	1.72	0.97	0.73	1.27
Oct	0.96	0.62	1.49	1.01	0.74	1.40	1.10	0.84	1.44
Nov	0.94	0.61	1.47	1.02	0.74	1.41	1.16	0.88	1.53
Dec	*1.60*	*1.03*	*2.49*	1.30	0.94	1.79	*0.72*	*0.55*	*0.95*
Public holiday^a^	1.01	0.61	1.68	1.26	0.86	1.84	1.04	0.75	1.43
*BOR* ^a^	*1.01*	*1.00*	*1.02*	*1.02*	*1.00*	*1.04*	1.01	0.99	1.02
*Admission rate* ^a^	*0.97*	*0.95*	*1.00*	0.99	0.97	1.00	*0.99*	*0.98*	*1.00*
BWT (95th percentile)^a^	1.13	0.98	1.29	1.03	0.99	1.08	0.99	0.97	1.01
*Time trend*	*1.00* ^b^	*1.00*	*1.00*	*1.00* ^b^	*1.00*	*1.00*	*1.00* ^c^	*1.00*	*1.00*

Significant factors were italicized

^a^Refers to the index visit to ED

^**b**^Decreasing trend

^c^Increasing trend

In order to see if the patients who visited on Sunday were more severe than the patients who visited on Thursday, we also generated data on patient acuity category (PAC), an indicator used by ED doctors to assess patients’ acuity level. PAC is a common triaging system used in all public hospitals in Singapore. It has four categories from P1 to P4, where P1 refers to patients of resuscitation, cardiovascular collapse, or imminent danger of collapse, required to be attended to without a moment's delay; P2 refers to patients of non-resuscitation, major emergency, or ill and non-ambulant or having severe symptoms and trolley based; P3 refers to patients of minor emergency or ambulant with mild to moderate symptoms; and P4 refers to patients of no emergency or ambulant with mild symptoms [25]. The patients who visited and discharged from ED on Sunday were actually less emergent or ambulant than the patients on Thursday. The total numbers of P1 and P2 patients on Sunday were slightly lower than those on Thursday in all three hospitals. The total percentages of P1 and P2 patients on Sunday compared with Thursday were 16.6 vs. 19.4 % in hospital A; 22.8 vs. 25.7 % in hospital B; and 32.3 vs. 36.4 % in hospital C (Table 4). PAC was not included as a confounding factor in our analyses due to the following reasons: first, PAC is a local parameter which may not be available in the EDs of other countries; and second, even in Singapore, the definition of PAC could hardly be standardized among different hospitals.

**Table 4:** Proportion of P1 and P2 patients among those who visited and were discharged properly from ED

Day of week	(P1 + P2)%		
	Hospital A	Hospital B	Hospital C
Mon	18.2	25.7	34.2
Tue	18.9	26.2	36.4
Wed	18.1	25.1	35.6
Thu	19.4	25.7	36.4
Fri	19.1	26.0	37.3
Sat	19.3	26.2	36.4
Sun	16.6	22.8	32.3

## Discussion

The average unplanned 3-day re-attendance rate in the three hospitals varied from 3.9 to 4.9 %, lower than 5 % set by many countries as a quality indicator [1, 2, 6]. Based on a rough review of the ICD diagnosis codes, about 40 % of the unplanned 3-day ED re-attendances were due to the same clinical diagnosis as the index ED attendance. Among the patients who revisited the ED due to different complaints, the top 10 diagnoses were acute upper respiratory infections; abdominal pain; gastroenteritis, colitis, or gastritis; pyrexia; headache, dizziness, or giddiness; chest pain; cellulitis or abscess; pneumonia; and infectious and parasitic diseases. Further studies need to be carried out to explore if there are any problem with current discharge process or post-discharge follow-up.

The association between BOR and the ED re-attendance within 3 days in hospitals A and B was low (risk ratio of 1–2 %). The tight bed situation caused by the higher BOR could result in a higher threshold of the patients being discharged from ED in a more serious state, i.e., patients who need to be admitted were actually discharged from ED. However, the association between BOR and ED re-attendance was not significant for hospital C. This is because hospital C has consistently operated at its upper limit of daily BOR, and a small increase in BOR did not affect the ED re-attendance rate. The result might indicate that despite the tight bed situation, quality of care at the ED as measured by the 3-day unplanned re-attendance rate has not been compromised.

A higher ED admission rate was associated with lower 3-day re-attendance rate, an expected finding. This could be that as a higher proportion of patients were admitted for inpatient care, the discharged patients were relatively less severe resulting in a lower likelihood of re-attendance to ED within 3 days. Longer bed waiting time was not associated with ED re-attendance rate neither. It might indicate that the quality of the services delivered at ED as measured by the 3-day unplanned re-attendance rate was not affected by the crowding in ED caused by patients waiting longer time to be admitted to wards.

There was no difference in the unplanned 3-day re-attendance rate for the patients who visited ED on public holidays compared to those who visited on other days. However, there was a strong day-of-the-week effect on ED re-attendance rate. The patients who visited ED on Thursday were least likely to re-attend the ED within 3 days (Friday–Sunday); the patients who visited on Sunday were most likely to re-attend the ED within 3 days (Monday–Wednesday). The average age of the patients who visited on Thursday was about 5 years older than that of the patients who visited on Sunday. This could be due to the closure of most of primary care centers during weekends, especially on Sundays. The patients who visited on Sunday were also less emergent or ambulant than the patients who visited on Thursday. The patients were less likely to revisit ED on Friday to Sunday, which could be caused by characteristics at both patients’ level and hospitals’ level [26]. Patients’ characteristics might include the following: lower stress levels on weekends may reduce symptoms of illness [27, 28]; patients’ care needs could be taken cared by their family members or friends who are more likely to be available on weekends [27, 28]; the patients had less incentive to re-visit to ED for medical certificate (MC) on weekends (primary studies are needed to confirm this hypothesis). MC issued by a medical doctor is generally required for taking sick leave in Singapore. Hospital level characteristics could include disparities in resource allocation or operational management on different days of week. Less senior doctors or nurses work on Sunday; some tests, treatments, or therapies may not be available on Sunday, the patients were less likely to be followed up timely or appropriately due to the close of most primary or specialist care centers [29, 30]. More research into this area needs to be done in the future.

There were a few limitations in this study. First, some important factors which may affect the ED re-attendance rate were not adjusted in the study, like the staffing level at ED, experience and skill levels of ED physicians, patient severity, etc. These factors were hard to measure, especially at daily level. Another limitation is that patients making an unplanned revisit to EDs not in hospitals A, B, or C were not counted, but empirical knowledge suggests that this proportion is very small.

## Conclusions

A study using time series data has been conducted to study factors associated with the unplanned 3-day ED re-attendance rate, and especially if higher BOR was associated with higher ED re-attendance rate in Singapore. The association between BOR and 3-day ED re-attendance rate varied with hospital. Strong day-of-week effect was first reported. Further research need to understand the reasons behind.
